# Role of the NC-Loop in Catalytic Activity and Stability in Lipase from *Fervidobacterium changbaicum*

**DOI:** 10.1371/journal.pone.0046881

**Published:** 2012-10-08

**Authors:** Binchun Li, Guangyu Yang, Lie Wu, Yan Feng

**Affiliations:** 1 State Key Laboratory of Microbial Metabolism, School of Life Sciences and Biotechnology, Shanghai Jiao Tong University, Shanghai, P. R. China; 2 Key Laboratory for Molecular Enzymology and Engineering of Ministry of Education, Jilin University, Changchun, P. R. China; University of Graz, Austria

## Abstract

Flexible NC-loops between the catalytic domain and the cap domain of the α/β hydrolase fold enzymes show remarkable diversity in length, sequence, and configuration. Recent investigations have suggested that the NC-loop might be involved in catalysis and substrate recognition in many enzymes from the α/β hydrolase fold superfamily. To foster a deep understanding of its role in catalysis, stability, and divergent evolution, we here systemically investigated the function of the NC-loop (residues 131–151) in a lipase (FClip1) from thermophilic bacterium *Fervidobacterium changbaicum* by loop deletion, alanine-scanning mutagenesis and site-directed mutagenesis. We found that the upper part of the NC-loop (residues 131–138) was of great importance to enzyme catalysis. Single substitutions in this region could fine-tune the activity of FClip1 as much as 41-fold, and any deletions from this region rendered the enzyme completely inactive. The lower part of the NC-loop (residues 139–151) was capable of enduring extensive deletions without loss of activity. The shortened mutants in this region were found to show both improved activity and increased stability simultaneously. We therefore speculated that the NC-loop, especially the lower part, would be a perfect target for enzyme engineering to optimize the enzymatic properties, and might present a hot zone for the divergent evolution of α/β hydrolases. Our findings may provide an opportunity for better understanding of the mechanism of divergent evolution in the α/β hydrolase fold superfamily, and may also guide the design of novel biocatalysts for industrial applications.

## Introduction

The α/β hydrolase fold superfamily is one of the largest structurally related protein groups in nature [1]. The enzymes in this superfamily can recognize a broad variety of functional groups, including carboxylic acids, epoxides, peptides, alkyl halides, and hydroxynitriles [2]. For this reason, they are one of the most widely used groups of biocatalysts in industry [3]. However, although the members of the α/β hydrolase fold superfamily display great diversity in their sequence and function, all of them have a canonical α/β hydrolase fold, which consists of a mostly parallel, twisted, eight-stranded β-sheet flanked by α-helices on both sides. This core architecture functions as a stable scaffold to accommodate the catalytic residues and can tolerate large insertions without losing its structure or catalytic machinery. The insertions can occur at several locations, including the loops after β3, β4, β6, β7 or β8 [4]. They can vary from a few amino acid residues to a complete extra domain. This affects the geometry and microenvironment of the active site, and gives the family members striking abilities to accommodate different substrates.

The insertion after the β6 sheet is the most important one in the α/β hydrolase fold superfamily. It forms the lid or cap domain of many family members [2], [4], and has great impact on the substrate recognition and catalysis [5], [6]. Recently, it has been found that the flexible loops that link the insertions onto the core architecture also play important roles in enzymatic function. The loop, the so-called NC-loop, is located right after the β6 sheet, and it connects the N-terminus of the catalytic domain and the cap domain. After studying the sequence and structure of epoxide hydrolases, Pleiss and his colleagues proposed that NC-loop might interact with the substrate by defining the substrate-binding pocket and regulating the accessibility of active site [7], [8]. Later, it was proved that the residue Tyr on the NC-loop of epoxide hydrolases is involved in substrate binding, stabilization of the transition state, and possibly protonation of the epoxide oxygen [9], [10]. The NC-loop of the esterase ybfF carries the catalytic residue Asp [11]. The catalytic residue Glu of haloalkane dehalogenases is also found within the NC-loop [12]. All these evidences suggest that exploring the functional and evolutionary role of the NC-loop may be important for understanding the adaptation and evolution of these kinds of enzymes.

Lipases prefer to hydrolyze water-insoluble triglycerides to glycerol and fatty acids. They have tremendous potential applications in the synthesis of optically active compounds, bulk products such as laundry detergents, lipids and biodiesel [13]. The lipase V family is a subfamily of the bacterial lipolytic enzyme family. The enzymes belonging to this subfamily share certain amino acid sequence similarity (20–25%). Besides lipases, there are also various bacterial non-lipolytic enzymes in this family, such as epoxide hydrolases, dehalogenases, meta-cleavage product hydrolases, and non-heme haloperoxidases (perhydrolases) [14]. The members of the lipase V family have structures consisting of an α/β hydrolase fold domain and a cap domain. A long NC-loop linking these two domains participates in the formation of the substrate-binding site and may modulate the movement of the cap domain. The NC-loops of epoxide hydrolases and haloalkane dehalogenases have been proved to play important roles in enzyme catalysis and substrate binding, but little attention has been paid to the functional role of the NC-loop in the lipase V family.

**Figure 1:**
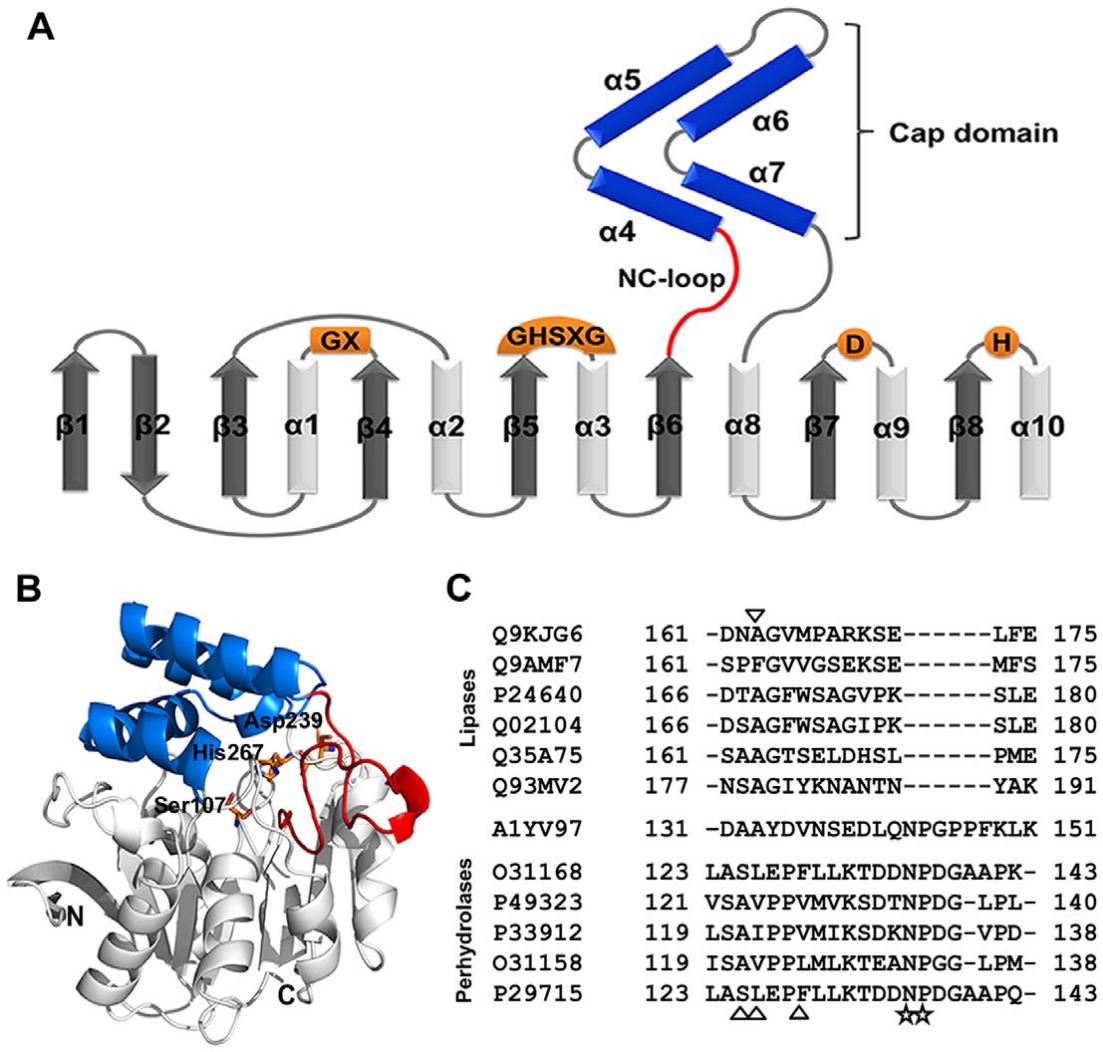
The structure of FClip1 and sequence alignment of FClip1 with related α/β hydrolases in the NC-loop region. (A) Secondary structure topology diagram of FClip1. The α-helices and β-sheets are represented by cylinders and arrows, respectively. The α/β hydrolase fold domain is shown in gray and white, the cap domain is shown in blue, and the NC-loop is shown in red. The locations of the conserved residues of the catalytic machinery are shown in orange. (B) The modeled structure of FClip1. The catalytic triad Ser107, His267, and Asp239 are shown in ball and sticks. (C) Sequence alignment of FClip1 with other lipases and perhydrolases in the NC-loop region. The residues of other lipases similar to FClip1 are indicated by the inverted triangles. The residues of the perhydrolases similar to FClip1 are indicated by triangles. The highly conserved residues are indicated by stars. Q9KJG6: lipase lip3 from *Pseudomonas aeruginosa* LST-03; Q9AMF7: triacylglycerol acyl hydrolase from *Moritella marina*; P24640: lipase lip3 from *Moraxella* sp. TA144; Q02104: triacylglycerol lipase from *Psychrobacter immobilis*; Q35A75: probable lipase from *Bradyrhizobium* sp. BTAi1; Q93MV2: lipase from *Streptococcuss* sp. N1; A1YV97: lipase FClip1 from *Fervidobacterium changbaicum*; O31168: non-haem chloroperoxidase from *Streptomyces aureofaciens*; P49323: chloroperoxidase from *Streptomyces lividans*; P33912: bromoperoxidase BPO-A1 from *Streptomyces aureofaciens*; O31158: non-heme chloroperoxidase from *Pseudomonas fluorescens*; P29715: bromoperoxidase BPO-A2 from *Streptomyces aureofaciens*.

Recently, we cloned a novel lipase gene from thermophilic bacterium *Fervidobacterium changbaicum*. This lipase belongs to the lipase V family. The gene was overexpressed in *E. coli* and the recombinant protein FClip1 showed high activity towards triglycerides and *p*-nitrophenyl esters with varying alkyl chain lengths [15]. FClip1 displayed extreme stability at high temperatures, and had a maximum activity at 80°C. Structural modeling suggested that it has a canonical α/β hydrolase fold domain and a cap domain. Its NC-loop has 21 amino acid residues and links the β6 strand of the catalytic domain to the α4 helix of the cap domain (Figure 1A and 1B). This loop is longer than its counterparts in the lipase V family but shows considerable similarity to that of the perhydrolase family (Figure 1C). This implies that it might have some unique function in this enzyme and evolutionary relevance to the perhydrolase family.

In this paper, the function of NC-loop in FClip1 was systemically studied by loop deletion, alanine-scanning mutagenesis and site-directed mutagenesis. Further insight into the mechanism was gained by kinetic analysis and biophysical characterization of the mutants. Together with structural modeling, we demonstrated the functional significance of the NC-loop in catalysis and stability. We found that the NC-loop, especially its lower part, could tolerate extensive modification and was highly plastic, making it a suitable target for enzyme engineering.

## Results and Discussion

### Structural Features of the NC-loop in FClip1

FClip1 is a new member of lipase V family. In order to obtain its structural information, the modeled structure of FClip1 was constructed by using Phyre 2 Protein Fold Recognition Server with the aryl esterase PFE from *Pseudomonas fluorescens* (PDB code: 1VA4) as a template (see Table S1, Figure S1 and S2A for the details). Phyre 2 uses the alignment of hidden Markov models and incorporates *ab initio* folding simulation. This significantly improves modeling accuracy that could routinely create the accurate models at very low sequence identity (15–25%) [16]. Phyre 2 has been used successfully to model the 3D structures of many proteins, such as esterase [17], endonuclease [18] and phosphomevalonate kinase [19], providing valuable and timely information for both basic research and industrial applications. The modeled structure of FClip1 possesses a catalytic domain and a cap domain. In this way, it is similar to the epoxide hydrolases, haloalkane dehalogenases, meta-cleavage product hydrolases and non-heme haloperoxidases of the α/β hydrolase fold superfamily (Figure 1A and 1B). The catalytic domain of FClip1 has a canonical α/β hydrolase fold and the active site is located at the interface between the two domains. The cap domain is constituted of four α-helices and locates on the top of the catalytic domain.

Accurately defining the boundary of NC-loop is crucial for studying its structure-function relationship. The modeled structure of FClip1 shows that the NC-loop contains 21 residues (Asp131–Lys151), and connects the β6 strand of the catalytic domain and the α4 helix of the cap domain. In order to further validate the recognition of NC-loop, the secondary structure of FClip1 was predicted independently as a complement to the 3D structure modeling. The amino acid sequence of FClip1 was submitted to the online servers Porter [20] and SCRATCH [21], respectively. Consistent with the modeled structure, the results of both servers suggested that the NC-loop connected the catalytic domain and the cap domain, and was a long loop region starting from the residue Asp131 (Figure S3).

**Figure 2:**
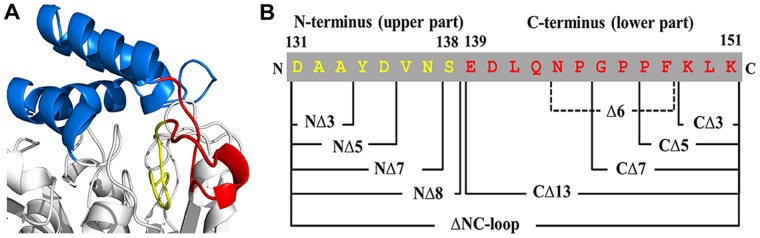
Design of the NC-loop deletion mutagenesis of FClip1. (A) Detailed structural conformation of the NC-loop. The upper part of the NC-loop (Asp131–Ser138), which is buried inside the protein, is shown in yellow; the lower part (Glu139–Lys151), which is exposed in the solvent, is shown in red. (B) The design of systematic deletion of the NC-loop.

As shown in Figure 1, the NC-loop demarcates the boundary of the two domains and performs a hinge-like function. It has been reported that such hinge-like loops usually regulate the relative orientation of two domains and play roles in modulating enzymatic activity and substrate specificity [22–24]. Based on the solvent accessibility surface of amino acid residues, NC-loop can be roughly divided into two parts (Figure 2A). The N-terminal part (Asp131–Ser138, upper part) is located in the interior of the protein and participates in the formation of the substrate-binding pocket, and the C-terminal part (Glu139–Lys151, lower part) is a highly surface-exposed region that contains relatively high numbers of charged residues (Glu139, Asp140, Lys149, and Lys151, Figure 2B).

Comparing the amino acid sequence of the NC-loop in FClip1 to its counterparts in the lipase V family revealed a six-residue insertion (Asn143–Phe148) in the middle of the NC-loop (Figure 1C). Given the fact that FClip1 is a thermophilic protein and is much more stable than other members of the lipase V family, this long NC-loop is an unusual feature because longer flexible loops are entropically more costly and thermodynamically unfavorable than short ones [25], [26]. Interestingly, we found that this NC-loop showed certain similarity to that of the perhydrolase family (Figure 1C). They share similar length (20–21 residues) and have two conserved residues (Asn143 and Pro144) in the C-terminal region of the NC-loop. Actually, phylogenetic tree analysis showed that FClip1 has the nearest evolutionary distance to the perhydrolase family among all the members from lipase V family (Figure S4). In addition, a trace of perhydrolase activity (about 3 mU/mg) was found in FClip1, which is about three orders of magnitude lower than its lipase activity (Li *et al*. unpublished results). We therefore speculated that FClip1 might be an evolutionary intermediate between perhydrolases and lipases.

### Effects of the NC-loop on the Catalytic Activity of FClip1

In order to understand the function of the NC-loop in catalysis, the ΔNC-loop mutant lacking all 21 residues of the NC-loop and the Δ6 mutant lacking the insertion region (Asn143–Phe148) were constructed (Figure 2B). Both of the mutants can be expressed in soluble form in *E. coli* with reasonable expression levels (∼5 mg protein/g cell). Far-UV circular dichroism (CD) spectra showed that both of the mutants had well-folded secondary structures and remained a typical α-helix/β-sheet spectral signature (Figure S5), suggesting that the deletions from the NC-loop did not change the overall folding state of the enzyme.

Enzymatic activities of the mutants were measured at 75°C using *p*NPC4 and *p*NPC12 as substrates, respectively. As shown in Table 1, deletion of the entire NC-loop (ΔNC-loop mutant) caused a complete loss of catalytic activity. Kinetic parameters showed that the reduction in enzymatic activity was mainly caused by a sharp decline of the *k*_cat_ value but not the *K*_m_ value (Table 1). The *k*_cat_ value of the ΔNC-loop mutant decreased by 42-fold relative to wild type (Table 1). This clearly indicates the importance of the NC-loop. The *K*_m_ value of the ΔNC-loop increased by 2.3-fold, suggesting that the NC-loop also affects the substrate binding affinity. This is consistent with the other α/β hydrolases that NC-loop participates in the formation of the substrate binding pocket, for example, in epoxide hydrolases [7], [8] and meta-cleavage product hydrolases [27], [28]. To our surprise, the Δ6 mutant retained about 60% of the activity of the wild type, indicating that the removal of the six-residue insertion did not have much effect on catalysis (Table 1). Its *k*_cat_ value decreased only slightly and its *K*_m_ value decreased by about 23%, resulting in a catalytic efficiency (*k*_cat_/*K*_m_) very similar to that of wild type.

**Table 1:** Specific activities and kinetic parameters of the wild type FClip1 and the deleted mutants.

Enzyme	Specific activity (U mg^−1^)		Kinetic parameters for *p*NPC4		
	*p*NPC4	*p*NPC12	*k*_cat_ (s^−1^)	*K*_m_ (mM)	*k*_cat_/*K*_m_ (mM^−1^s^−1^)
Wild type	5.8 (100.0)^a^	3.8 (100.0)^a^	54.5±2.0	1.3±0.1	41.9
ΔNC-loop	0.09 (1.6)	0.05 (1.3)	1.3±0.1	3.0±0.3	0.4
Δ6	3.4 (58.6)	2.5 (65.8)	39.4±1.8	1.0±0.1	39.4
NΔ3	0.06 (1.0)	0.03 (0.8)	0.5±0.03	2.1±0.2	0.2
NΔ5	0.01 (0.2)	0.01 (0.3)	0.3±0.03	3.6±0.6	0.1
NΔ7	0.05 (0.9)	0.01 (0.3)	1.6±0.4	6.2±2.1	0.3
NΔ8	0.08 (1.4)	0.03 (0.8)	1.2±0.2	3.2±0.7	0.4
CΔ3	9.9 (170.7)	3.6 (94.7)	74.9±2.8	1.1±0.1	68.1
CΔ5	8.2 (141.4)	3.3 (86.8)	71.5±2.5	1.2±0.1	59.6
CΔ7	7.3 (125.9)	2.5 (65.8)	64.5±3.3	1.3±0.1	49.6
CΔ13	12.7 (219.0)	3.8 (100.0)	105.7±5.0	1.2±0.1	88.1

Specific activities were measured in 50 mM phosphate buffer (pH 8.0) at 75°C using *p*NPC4 and *p*NPC12 as the substrates, respectively. Kinetic parameters were obtained in 50 mM phosphate buffer (pH 8.0) at 75°C using *p*NPC4 as the substrate. The fitting curves for kinetic parameters are presented in Figure S6.

a Numbers in brackets indicate the values relative to wild type.

Above results show the NC-loop plays crucial roles in catalytic process. However, the six-residue insertion can be deleted without damaging the activity. This suggests that the catalytic function of FClip1 is not related to this inserting region. In order to investigate the functional elements of the NC-loop in more detail, we designed and constructed a series of mutants by systematically truncating the NC-loop from its N-terminal or C-terminal (Figure 2B). All the mutants could be expressed in soluble form in *E. coli*, and the far-UV CD spectra of the representative mutants show that the overall folding states of the enzymes were not affected by the deletions from the NC-loop (Figure S5). However, the deletions at different regions of NC-loop resulted in different structural consequences. For the NΔ8 mutant, the mean residue ellipticity ([θ]_MRW_) at 193 nm lowered and the minima at 208 and 222 nm lost intensity compared to wild type. The calculation of secondary structure composition showed the helical content of the NΔ8 mutant decreased by 11% (Table S2). For the CΔ7 and CΔ13 mutants, there was a substantial increase in helical structure by 8% and 6%, respectively, as indicated by the increased signals at 193, 208 and 222 nm.

Deletions from the N-terminal and C-terminal of the NC-loop showed very different effects on enzymatic activity. Deletions from the upper part of the NC-loop (NΔ3, NΔ5, NΔ7, and NΔ8 mutants) caused the enzyme to lose more than 98.6% of its activity (Table 1). Removing as little as three residues could greatly inactivate the enzyme. The deletions caused slight increases in *K*_m_ and dramatic decreases in *k*_cat_, whose effects are similar to those of the removal of the entire NC-loop (Table 1). The lower part of the NC-loop (Glu139–Lys151), however, was able to tolerate extensive deletion without loss of activity. The CΔ3, CΔ5, CΔ7, and CΔ13 mutants showed comparable activity for *p*NPC12 to that of the wild type enzyme, and increased activity for *p*NPC4 (Table 1). The CΔ13 mutant, which lacked all 13 residues of the lower part (Glu139–Lys151), even increased its catalytic efficiency about 2.1-fold. Deletions from the lower part of the NC-loop were also found to change the substrate preference. The CΔ3, CΔ5, CΔ7, and CΔ13 mutants were more favorable to *p*NP-esters with shorter acyl chain length than the wild type enzyme. The selectivity of the CΔ3, CΔ5, CΔ7, and CΔ13 mutants between *p*NPC4 and *p*NPC12 were increased 1.8-, 1.6-, 1.9-, and 2.2-fold, respectively. This confirmed our structural modeling results that the NC-loop is involved in substrate binding.

In order to pinpoint the residues that might account for the loss of activity of the upper part deletion mutants, we performed alanine-scanning mutagenesis on the upper part of the NC-loop. The residues within the Asp131-Ser138 region were substituted with Ala except for Ala132 and Ala133. All mutants retained a considerable degree of activity, no less than 70% of that of wild type (Table 2). The best mutant D131A even enhanced the specific activity about 2.2-fold and exhibited a catalytic efficiency (*k*_cat_/*K*_m_) 1.8-fold higher than that of wild type. These results indicated that the residues in this region are not directly involved in enzyme catalysis. Considering this and the fact that the lower part of the NC-loop can be deleted without loss of activity, we conclude that none of the side chains on the NC-loop is essential to the catalysis. This is different from some members of the α/β hydrolase fold superfamily, in which the residues on the NC-loop play crucial roles in the catalytic process, such as stabilizing the transition state [9], anchoring the substrate [10], or serving as the catalytic residue [11], [12]. NC-loop also serves the hinge-like function and its flexibility mediates the orientation of the cap domain. The variability in the position of the cap domain is likely to have a significant impact on activity and substrate recognition [22]. Therefore, the NC-loop may be an important region to divergent evolution in the α/β hydrolase fold superfamily. It has been reported that the NC-loop plays a crucial role in the conversion of an esterase into the epoxide hydrolase [29]. This is an excellent example of the evolutionary role of the NC-loop in the α/β hydrolase fold superfamily. In this way, the evolution of the NC-loop may have been an important event during the divergent evolution of α/β hydrolase fold superfamily.

**Table 2:** Specific activities and kinetic parameters of the wild type FClip1 and its mutants.

	Enzyme	Specific activity (U mg^−1^)	*k*_cat_ (s^−1^)	*K*_m_ (mM)	*k*_cat_/*K*_m_ (mM^−1^ s^−1^)
	Wild type	5.8 (100.0)^a^	54.5±2.0	1.3±0.1	41.9
Ala-scaning mutagenesis	D131A	13.0 (224.1)	74.3±3.6	1.0±0.1	74.3
	Y134A	4.1 (70.7)	30.6±0.9	1.0±0.1	30.6
	D135A	6.3 (108.6)	54.1±5.1	1.4±0.2	38.6
	V136A	7.0 (120.7)	56.4±3.7	1.5±0.2	37.6
	N137A	5.2 (89.7)	46.0±3.6	1.4±0.2	32.9
	S138A	6.0 (103.4)	54.4±4.3	1.5±0.2	36.3
Tyr134 saturation mutagenesis	Y134G	3.0 (51.7)	29.2±1.3	1.5±0.1	19.5
	Y134S	2.3 (39.7)	23.5±1.3	1.5±0.2	15.7
	Y134R	0.4 (6.9)	6.1±0.8	2.9±0.6	2.1
	Y134E	0.3 (5.2)	5.7±0.7	3.1±0.6	1.8
	Y134V	3.4 (58.6)	34.4±1.7	1.1±0.1	31.3
	Y134I	3.9 (67.2)	40.4±1.6	1.3±0.1	31.1
	Y134F	4.9 (84.5)	38.9±1.7	1.0±0.1	38.9

Enzyme assays were performed in 50 mM phosphate buffer (pH 8.0) at 75°C using *p*NPC4 as the substrate. The fitting curves for kinetic parameters are presented in Figure S6.

a Numbers in brackets indicate the values relative to wild type.

Although the residues on the NC-loop are not essential to the enzymatic reaction, many of them have the ability to fine-tune enzyme activity. Except for the inactive mutants (activity <1.5% that of wild type), many mutations on the NC-loop caused the changes of more than 10% in enzymatic activity (Table 1 and Table 2). The highest activity level was observed in the D131A and CΔ13 mutants, which both showed 2.2-fold higher activity than that of the wild type. In contrast, the mutations at Tyr134 could result in lower activities than the wild type (Table 2). As a result, the NC-loop mutations give the enzyme considerable plasticity in fine-tuning its catalytic activity. For example, the *k*_cat_ values of the mutants ranged from 5.7 s^−1^ (Y134E) to 74.3 s^−1^ (D131A), about 10% and 136% of that of wild type, respectively. The *K*_m_ values of the mutants ranged from 1.0 mM (D131A) to 3.1 mM (Y134E), about 77% and 238% of that of wild type, respectively. As a consequence, the catalytic efficiency (*k*_cat_/*K*_m_) of the mutant Y134E was 41-fold lower than that of the best mutant D131A. This indicates that the NC-loop may play a regulatory role in the catalytic process, and can be used as a plastic region to be further engineered to optimize the enzymatic activity.

### Effects of Truncation of the NC-loop on the Stability of FClip1

Deletion mutations were studied to determine their influence on the thermostability (Table 3 and Figures S7–S9). The mutants lacking some residues in the lower part of NC-loop showed the enhanced thermostability in thermal inactivation experiments (Figure S7 and Table 3). When longer portions of the lower part were removed from this region, the thermostability of the mutants was gradually increased. For example, the CΔ3 mutant saw a 3.1-fold increase in half-life (*t*_1/2_) at 78°C relative to wild type. Increases of 4.4-, 8.8-, and 11.9-fold were observed for the CΔ5, CΔ7, and CΔ13 mutants, respectively (Table 3). In addition, temperature-activity curves showed that the deleted mutants had higher optimal temperatures (*T*_opt_), which correlated well with the results of thermal inactivation experiments (Figure S8 and Table 3). In order to obtain further information on the thermodynamic stability, thermal unfolding experiments of wild type and the deleted mutants were carried out by differential scanning calorimetry (DSC) (Figure S9). As shown in Table 3, the melting temperatures (*T*_m_) of the CΔ3, CΔ5, CΔ7, and CΔ13 mutants were 1.5, 1.7, 2.8, and 3.8°C higher than that of wild type, respectively. Probably because of protein aggregation at high temperatures, rescanning of denatured samples did not show heat absorbance, indicating that the thermal unfolding of wild type and the deleted mutants were irreversible.

**Table 3:** Thermodynamic parameters of the wild type FClip1 and the NC-loop deletion mutants.

	Thermal unfolding			GdnHCl-induced unfolding						
Enzyme	*T*_opt_ (°C)	*t*_1/2, 78°C_ (min)	*T*_m_ (°C)	*C*_50_ (M)	ΔG_1_ (kJ mol^−1^)	m_1_ (kJ L mol^−2^)	*C*_m1_ (M)	ΔG_2_ (kJ mol^−1^)	m_2_ (kJ L mol^−2^)	*C*_m2_ (M)
Wild type	80.0	4.9 (1.0) a	79.0	0.6	–	–	–	35.4	9.1	3.9
Δ6	82.5	14.5 (3.0)	80.5	0.7	–	–	–	29.3	7.5	3.9
CΔ3	82.5	15.4 (3.1)	80.5	0.7	–	–	–	31.5	8.1	3.9
CΔ5	82.5	21.8 (4.4)	80.7	0.8	–	–	–	31.6	8.1	3.9
CΔ7	82.5	43.0 (8.8)	81.8	0.8	–	–	–	30.4	7.8	3.9
CΔ13	82.5	58.1 (11.9)	82.8	1.0	22.9	16.3	1.4	21.8	5.8	3.8

a Numbers in brackets indicate the values relative to wild type.

The chemical stabilities of the wild type FClip1 and the deleted mutants were investigated by monitoring guanidine hydrochloride (GdnHCl)-induced inactivation (Figure S10 and Table 3). As in the thermostability tests, the deleted mutants exhibited better chemical resistance than wild type. After treatment with 1.2 M of GdnHCl for 2 h, wild type retained only 28% of its original activity, but the deleted mutants retained about 32–54% of their original activities (Figure S10). The *C*_50_ value, which indicates the concentration of GdnHCl capable of inhibiting 50% of the enzymatic activity, increased by 0.1, 0.2, 0.2, and 0.4 M for the CΔ3, CΔ5, CΔ7, and CΔ13 mutants, respectively. The more residues were removed from the lower part of NC-loop, the more stability of the active site was observed.

However, GdnHCl-induced protein unfolding/refolding experiments showed different results. The protein unfolding and refolding processes of all the proteins were highly reversible (Figure S11). As shown in Table 3, the deleted mutants had similar *C*_m2_ values with wild type, suggesting that the deletions from the lower part of NC-loop did not change the overall rigidity of the protein structure. Together with the increased thermostabilty and chemical resistance, these results suggest that the stabilizing effect of the loop-deletion mutants may come from the increased local stability of the active site. The lower part of the NC-loop might be highly flexible, thus the deletions from this region could increase the local rigidity of the protein. Given the participation of the NC-loop in the formation of substrate pocket, it is not surprising that the deletions from the NC-loop would affect the flexibility of the active site. This is consistent with previous bioinformatic surveys and accumulated structural studies showing that shorter loop regions are a common strategy for the proteins to improve the stability [30]–[32].

### Insight into the Structural Changes Caused by Deletion of the Lower Part of NC-loop

**Figure 3:**
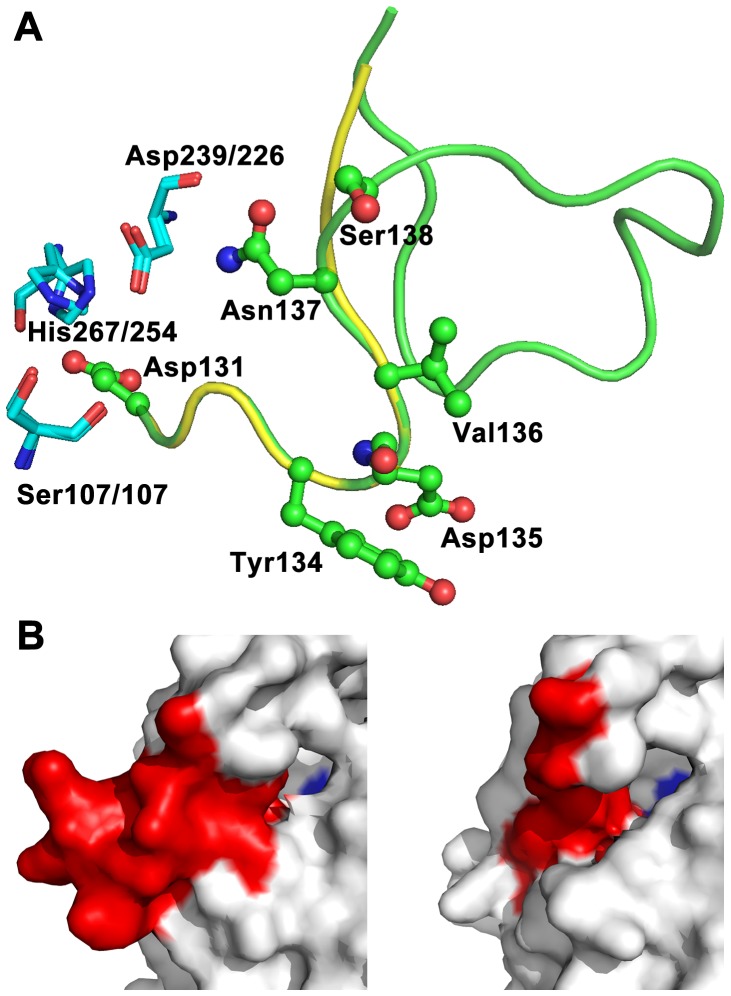
Structural comparison of the wild type FClip1 and the CΔ13 mutant. (A) Structural differences between the wild type FClip1 and the CΔ13 mutant near the NC-loop region. The NC-loop of the wild type FClip1 is shown in green and that of the CΔ13 mutant is shown in yellow. The catalytic triads are shown in cyan, thick lines. The residues subjected to site-directed mutagenesis are shown in ball and sticks. (B) The entrance of the substrate binding pocket of the wild type FClip1 (left) and the CΔ13 mutant (right). The surface of NC-loop is shown in red, and the catalytic residue Ser107 is shown in blue.

The CΔ13 mutant is a special mutant, which displayed a 2-fold increase in activity and a 12-fold increase in stability over wild type. In other word, there is no compromise between function and stability for this mutant. This is an unusual phenomenon because it does not obey the generally accepted principle of stability-function tradeoffs [33]. This kind of mutations is very rare in point mutations and may be valuable for protein engineering. In order to better understand the structural basis of this property, the 3D model of CΔ13 was constructed by homology modeling using the above-mentioned wild type structure as a template (see Figure S2B for Ramachandran plot of the model). The structure generated for the CΔ13 mutant was very similar to that of wild type. As shown in Figure 3A, the catalytic triads of the two enzymes overlap very well, except that the CΔ13 mutant possesses a much shorter NC-loop than the wild type. The secondary structure composition of the modeled structures of wild type and the CΔ13 mutant was similar to that calculated from CD spectroscopy (Table S2), suggesting that our structural modeling was relatively accurate.

The lower part (Glu139-Lys151) of NC-loop located near the entrance of the substrate-binding pocket and might participate in enzyme-substrate interaction by providing Phe148 and Leu150 as the sidewall of the pocket. Therefore, any modification at this region may have significant impact on the geometry of the substrate-binding pocket. As shown in Figure 3B, deletion of the loop changed the shape and enlarged the entrance of the substrate-binding pocket. The electrostatic potential of the protein surface near the NC-loop also changed significantly because of the removal of four charged residues (Glu139, Asp140, Lys149, and Lys151). These structural differences altered the microenvironment of the active site, which may account for the change in the activity of the CΔ13 mutant.

The structural information allowed us to expound the changes in the properties of the CΔ13 mutant. On one hand, CΔ13 had a much shorter NC-loop that decreased the redundant flexibility near the active site, and thus increased the stability of the enzyme. On the other hand, deletion of the lower part of NC-loop changed the geometry and electrostatic distribution of the substrate-binding pocket, which might account for the change in activity. This kind of structural effects is rare to achieve by regular site-directed mutagenesis. Actually, the importance of loop deletions and insertions has been well recognized in both natural protein evolution and protein engineering [34–36]. The CΔ13 mutant improved activity and stability simultaneously, presenting a good example to demonstrate the evolutionary potential of NC-loop. Given the great divergence of NC-loop in the different α/β hydrolases, this region seems to have great potential to be evolved for a particular functional requirement.

### Conclusion Remarks

Flexible loops play important roles in many protein functions, such as hinging the concerted movement between protein domains [37], participating in the formation of the binding sites for metallic cations [38] and ligands [39], mediating protein-protein interactions [40], and modulating protein stability [41]. The insertions and deletions of loop regions on protein scaffolds are the key event during the divergent evolution of enzyme families [2]. In this study, we have systemically investigated the function of the NC-loop of FClip1 by loop deletion, alanine-scanning mutagenesis and site-directed mutagenesis. Our results suggest that the NC-loop could modulate both catalytic activity and stability of FClip1. While the upper part of the NC-loop is crucial to enzyme catalysis, the lower part can tolerate extensive modifications and may serve as a plastic region for further protein engineering. To our knowledge, this is the first study to thoroughly evaluate the role of the NC-loop in enzyme catalysis, stability, and divergent evolution. Because of the great diversity of the NC-loop in sequence, conformation, and function in the α/β hydrolase fold superfamily, we believe it is necessary to establish the panoramic sequence-structure-function relationship map of NC-loop in different α/β hydrolases. This will not only help us to better understand the mechanism of divergent evolution of the α/β hydrolase superfamily, but may also promote the engineering of this important group of enzymes for industrial applications.

## Materials and Methods

### Enzymes, Bacterial Strains, and Reagents

DNA polymerase and PCR reagents were purchased from TaKaRa (Otsu, Japan). Oligonucleotide primers were synthesized by BioBasic (Shanghai, China). Restriction enzyme Dpn I was obtained from Fermentas (Burlington, Canada). *E. coli* strain BL 21-CodonPlus (DE3)-RIL was used for gene expression. The substrates *p*-nitrophenyl butyrate (*p*NPC4), *p*-nitrophenyl caprylate (*p*NPC8), and *p*-nitrophenyl laurate (*p*NPC12) used for enzyme activity assays and kinetics were purchased from Sigma (St. Louis, U.S.). Other reagents were of analytical grade.

### Homology Modeling of FClip1

The Phyre 2 Protein Fold Recognition Server (http://www.sbg.bio.ic.ac.uk/phyre/) was used to derive the 3D structure of FClip1 (UniProt accession: A1YV97). The aryl esterase PFE from *Pseudomonas fluorescens* (PDB code: 1VA4) [42] was chosen as the template. Structural qualities of the generated models were evaluated using Protein Structure Validation Software [43] (PSVS, http://psvs-1_4-dev.nesg.org/). The structural integrities of the models were visually examined by PyMOL [44]. PyMOL was also used to generate images of the structures.

### Mutagenesis, Expression and Purification of the Enzymes

Site-directed and loop-deletion mutants of FClip1 were constructed using the QuikChange Mutagenesis protocol [45] on the recombinant plasmid pET-15b-FClip1 carrying the mature gene of FClip1. The primers used to generate the mutants are listed in Table S3. Mutagenic PCR was performed under the following conditions: 95°C for 4 min; 20 cycles of 95°C for 1 min, 55°C for 1 min and 72°C for 7 min; and a final extension at 72°C for 30 min. The template was then eliminated by digestion with 5 U of Dpn I at 37°C for 4 h. The remaining PCR product was purified using a PCR clean-up kit (Axygen, U.S.). Each purified PCR product was transformed into chemically competent cell *E. coli* strain BL21 CodonPlus (DE3)-RIL. Successful introduction of desired mutation was confirmed by DNA sequencing (BGI, China). The wild type FClip1 and its mutants were expressed and purified as described previously [15]. Protein concentration was determined using the Bradford method [46].

Far-UV circular dichroism spectra of FClip1 and its mutants were monitored by J-815 CD spectropolarimeter (JASCO, U.S.) from 190 to 250 nm at room temperature with the following parameters: protein concentration 0.1 mg ml^−1^, bandwidth 1.0 nm, data pitch 0.025 nm, cell length 0.1 cm, response time 1.0 s and scanning speed 50 nm min^−1^. Each scan was repeated three times and the average value was used for the analysis. The baseline was measured by using 50 mM phosphate buffer (pH 8.0). Spectra were corrected for buffer absorbance and converted to mean residue ellipticity ([θ]_MRW_). The secondary structure percentage was calculated by using the software CDPro [47] (CONTINLL, reference protein set: SMP56).

### Enzyme Activity Assay and Steady-state Kinetics

Enzymatic activities were determined by monitoring *p*-nitrophenol released at 405 nm using an UV-2550 spectrophotometer (Shimadzu, Japan) equipped with a thermal controller. Unless otherwise indicated, the standard assay was performed at 75°C in 50 mM phosphate buffer (pH 8.0) containing 100 µM of *p*-nitrophenyl ester (*p*NPC4 or *p*NPC12). One unit of enzymatic activity was defined as the amount of enzyme capable of releasing one µmol *p*-nitrophenol per minute. All the activity measurements were made in triplicate. The specific activity data presented in the results are the means obtained from at least two independent experiments.

Steady-state kinetics of the enzymes for *p*NPC4 were determined at 75°C in 50 mM phosphate buffer (pH 8.0). The initial rate of the catalysis reaction was measured with varied concentrations of *p*NPC4 (0.05–3.0 mM). Kinetic parameters V_max_ and *K*_m_ were acquired by fitting enzymatic activities as a function of substrate concentrations to the Michaelis-Menten equation using non-linear regression of the software GraphPad Prism 5.0. The parameter *k*_cat_ was obtained by using the equation *k*_cat_ = V_max_/[E], where [E] was the molar concentration of the enzymes.

### Thermal Stability and Thermal Unfolding

The effect of temperature on enzymatic activity was determined by measuring the enzymatic activities of FClip1 and its mutants within a temperature range of 50–90°C using *p*NPC8 as the substrate.

Thermal inactivation experiments of the wild type FClip1 and the mutants were performed by incubating the enzymes (0.5 mg ml^−1^) at 78°C for specified time intervals. The enzyme samples were then cooled on ice and the residual activity was measured in 50 mM phosphate buffer (pH 8.0) at 70°C using *p*NPC8 as the substrate. The first-order rate constant *k*_inact_ of thermal inactivation was obtained by plotting logarithmic percentages of residual activity against time of heat treatment. The half-life (*t*_1/2_) of thermal inactivation was calculated using the following equation (Eq. 1). The half-life data presented in the results are the means obtained from at least two independent experiments.
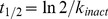
(1)


Differential scanning calorimetry (DSC) measurements were taked using MicroCal VP-DSC Differential Scanning Calorimeter (GE, U.S.). A protein concentration of approximately 1.0 mg ml^−1^ was used for the analysis. Denaturizing curves were recorded from 40°C to 90°C at a rate of 1°C min^−1^. The baseline scan was measured using 50 mM phosphate buffer (pH 8.0). The thermograms (C_p_ versus temperature) were analyzed using a two-state model in which the melting point *T*_m_ was fitted using MicroCal Origin software (OriginPro 7.0). The reversibility of thermal unfolding was evaluated by reheating the sample. The first scan was stopped at 85–87°C and cooled down to 40°C for the next consecutive scan. The experiments were performed twice and the data presented were the means of two measurements.

### Guanidine Hydrochloride-induced Unfolding

The enzyme inactivation induced by guanidine hydrochloride (GdnHCl) was performed by incubating approximately 0.12 mg ml^−1^ of proteins with a set of concentrations of GdnHCl for 2 h at room temperature. The residual activities of the enzymes were measured at 70°C using *p*NPC8 as the substrate in 50 mM phosphate buffer (pH 8.0) containing the corresponding concentration of GdnHCl. The *C*_50_ value, indicating the concentration of GdnHCl in which 50% of enzymatic activity was retained, was estimated by the same method as the calculation of *t*_1/2_.

Protein unfolding induced by GdnHCl was performed by incubating 0.1 mg ml^−1^ of proteins with a set of concentrations (0.0–6.0 M) of GdnHCl for 12 h at room temperature. The GdnHCl-induced unfolding for the wild type FClip1 and its mutants were monitored using a fluorescence spectrophotometer F-7000 (Hitachi, Japan) with an excitation wavelength of 290 nm at 25°C. The fluorescence spectra were recorded at the wavelengths between 300 and 400 nm at a scanning speed of 1200 nm min^−1^. The unfolding experiments were performed twice and the processed data were given as the means of two measurements. The data for wild type and the Δ6, CΔ3, CΔ5, and CΔ7 mutants were analyzed by two-state model (Eq. 2) using GraphPad Prism 5.0.
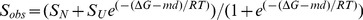
(2)Where S_obs_ is the observed spectroscopic signal, S_N_ and S_U_ are the signals for the native and unfolded species, respectively, ΔG is the free energy of unfolding in the absence of denaturant, m is the partial derivative of ΔG with respect to denaturant, and d is the concentration of denaturant. S_obs_ and d are the dependent and independent variables; S_N_, S_U_, ΔG, and m are treated as fitting parameters [48]. The *C*_m_, the GdnHCl concentration at the midpoint of GdnHCl-induced unfolding curve, was obtained using the following equation (Eq. 3).



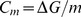
(3)Spectroscopic data for the CΔ13 mutant was analyzed by the three-state model (Eq. 4) using GraphPad Prism 5.0.
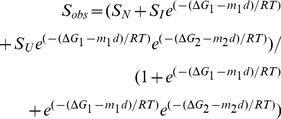
(4)Where S_obs_, S_N_, S_U_, and d have the same definitions as for the two-state model. ΔG_1_, ΔG_2_, m_1_, m_2_, S_N_, S_I_, and S_U_ are treated as fitting parameters. The *C*_m1_ and *C*_m2_ were obtained using the equations Eq. 5 and Eq. 6, respectively.
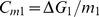
(5)

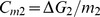
(6)


Protein refolding experiments were performed by mixing protein solutions with GdnHCl to the final concentration of 6.0 M GdnHCl and 1.2 mg ml^−1^ protein at room temperature for 12 h. This mixture was then diluted to different concentrations (0.5–6.0 M) of GdnHCl and 0.1 mg ml^−1^ protein. Spectral measurements were made after 12 h.

## Supporting Information

Figure S1 **Sequence alignment between FClip1 and the modeled template 1VA4.** The residues that are identical between query and template are highlighted in red, and the conserved and similar residues between query and template are highlighted in yellow. The NC-loop of FClip1 is underlined. The corresponding region in the template 1VA4 is also the NC-loop.
(TIF)

Figure S2 **Ramachandran plots of the modeled structures.** (A) Ramachandran plots of the modeled structure of the wild type FClip1. (B) Ramachandran plots of the modeled structure of the CΔ13 mutant.
(TIF)

Figure S3 **Secondary structure prediction for FClip1 by using the online servers Porter and SCRATCH.** H, E, and C in the prediction results stand for the secondary structure of helix, strand and coil, respectively. The NC-loop in the modeled structure of FClip1 is shown in red.
(TIF)

Figure S4 **Phylogenetic tree of the lipases and the perhydrolases (non-heme haloperoxidases).** P22862: aryesterase from *Pseudomonas fluorescens*; the other enzymes repesented by the accession numbers were the same as in figure 1. Although FClip1 (A1YV97) belongs to the lipase family, it situates at the root of the lipases branch and is near the perhydrolases branch. It might be an evolutionary intermediate between lipases and perhydrolases (Scale bar: 0.2 amino acid substitutions per site).
(TIF)

Figure S5 **Far-UV CD spectra of the wild type FClip1 and the NC-loop deletion mutants.** CD spectra were recorded from 190 to 250 nm. The spectra of wild type and the ΔNC-loop, Δ6, NΔ7, NΔ8, CΔ7, and CΔ13 mutants are shown in black, blue, red, pink, green, dark yellow, and grey, respectively. Protein concentration of 0.1 mg ml^−1^ was used for the analysis.
(TIF)

Figure S6 **Fitting curves for the kinetic parameters from non-linear regression methods.** Kinetic parameters V_max_ and *K*_m_ were acquired by fitting enzymatic activities as a function of substrate concentrations to the Michaelis-Menten equation using the software GraphPad Prism 5.0.
(TIF)

Figure S7 **Thermal inactivation profiles of the wild type FClip1 and the NC-loop deletion mutants.** After incubating the enzymes (0.5 mg ml^−1^) for specified time intervals at 78°C, the residual activities were measured in 50 mM phosphate buffer (pH 8.0) at 70°C using *p*NPC8 as the substrate. The data for wild type, the Δ6, CΔ3, CΔ5, CΔ7, and CΔ13 mutants are shown in ▪, ▾, ♦, ★, ▴, and •, respectively.
(TIF)

Figure S8 **Temperature dependence of enzymatic activity of the wild type FClip1 and the NC-loop deletion mutants.** Enzymatic activities were measured in the temperature range from 50 to 90°C using *p*NPC8 as the substrate. The data for wild type, the Δ6, CΔ3, CΔ5, CΔ7, and CΔ13 mutants are shown in ▪, ▾, ♦, ★, ▴, and •, respectively.
(TIF)

Figure S9 **Thermal unfolding of the wild type FClip1 and the NC-loop deletion mutants monitored by DSC scanning.** Protein concentration of 1.0 mg ml^−1^ was used for the analysis. Denaturizing curves were recorded from 40°C to 90°C with a rate of 1°C min^−1^. The data for baseline, wild type, and the Δ6, CΔ3, CΔ5, CΔ7, and CΔ13 mutants are shown in black, red, blue, olive, cyan, dark yellow, and pink, respectively.
(TIF)

Figure S10 **GdnHCl-induced inactivation of the wild type FClip1 and the NC-loop deletion mutants.** After 2 h of incubation with varied concentrations of GdnHCl at room temperature, the residual activities were measured in 50 mM phosphate buffer (pH 8.0) at 70°C using *p*NPC8 as the substrate. The data for wild type, the Δ6, CΔ3, CΔ5, CΔ7, and CΔ13 mutants are shown in ▪, ▾, ♦, ★, ▴, and •, respectively.
(TIF)

Figure S11 **GdnHCl-induced unfolding and refolding of the wild type FClip1 and the NC-loop deletion mutants monitored by fluorescence spectrophotometry.** The fluorescence spectra were recorded at the wavelengths between 300 and 400 nm with an excitation wavelength at 290 nm under a scanning speed of 1200 nm min^−1^. The measured data for protein unfolding and refolding are shown in • and ○, respectively. The fitted curves for protein unfolding were shown in solid lines.
(TIF)

Table S1 **Structural modeling information of FClip1 generated by the Phyre 2 server using different templates.** The qualities of the structures were evaluated by Protein Structure Validation Software (PSVS).
(DOCX)

Table S2 **Secondary structure analysis of the wild type FClip1 and the deleted mutants by CD spectra and structural modeling.**
(DOCX)

Table S3 **Oligonucleotide primers used in the mutagenic PCR for deletion mutagenesis and site-directed mutagenesis of FClip1.**
(DOCX)
