# Supplementary figures and images for: Role of the NC-Loop in Catalytic Activity and Stability in Lipase from Fervidobacterium changbaicum
Source: PLoS One. 2012 Oct 8;7(10):e46881. doi: 10.1371/journal.pone.0046881 (PMC3466181; doi:10.1371/journal.pone.0046881)

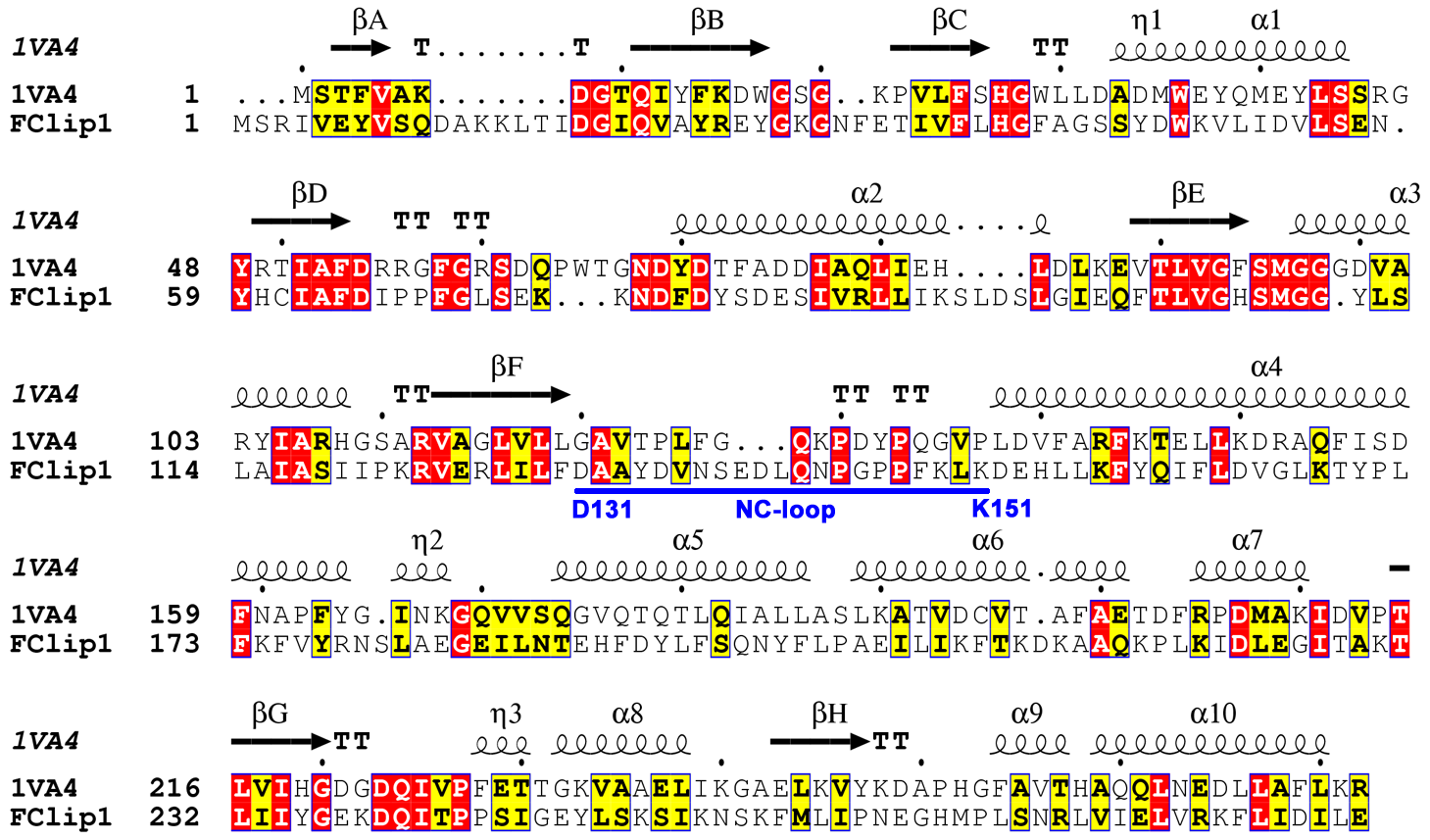

Supplement: Figure S1 — Sequence alignment between FClip1 and the modeled template 1VA4. The residues that are identical between query and template are highlighted in red, and the conserved and similar residues between query and template are highlighted in yellow. The NC-loop of FClip1 is underlined. The corresponding region in the template 1VA4 is also the NC-loop. (TIF) [file pone.0046881.s001.tif]

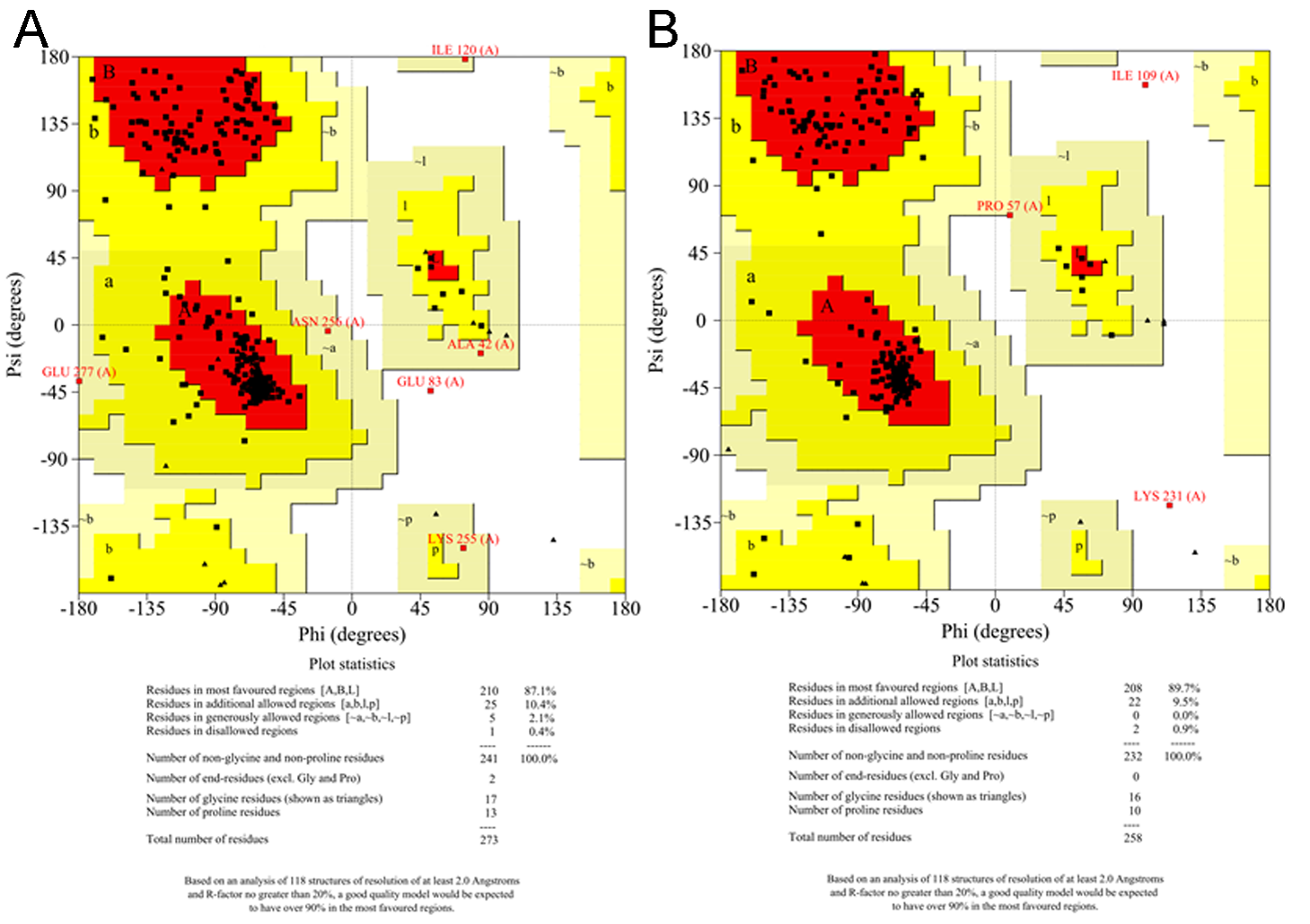

Supplement: Figure S2 — Ramachandran plots of the modeled structures. (A) Ramachandran plots of the modeled structure of the wild type FClip1. (B) Ramachandran plots of the modeled structure of the CΔ13 mutant. (TIF) [file pone.0046881.s002.tif]

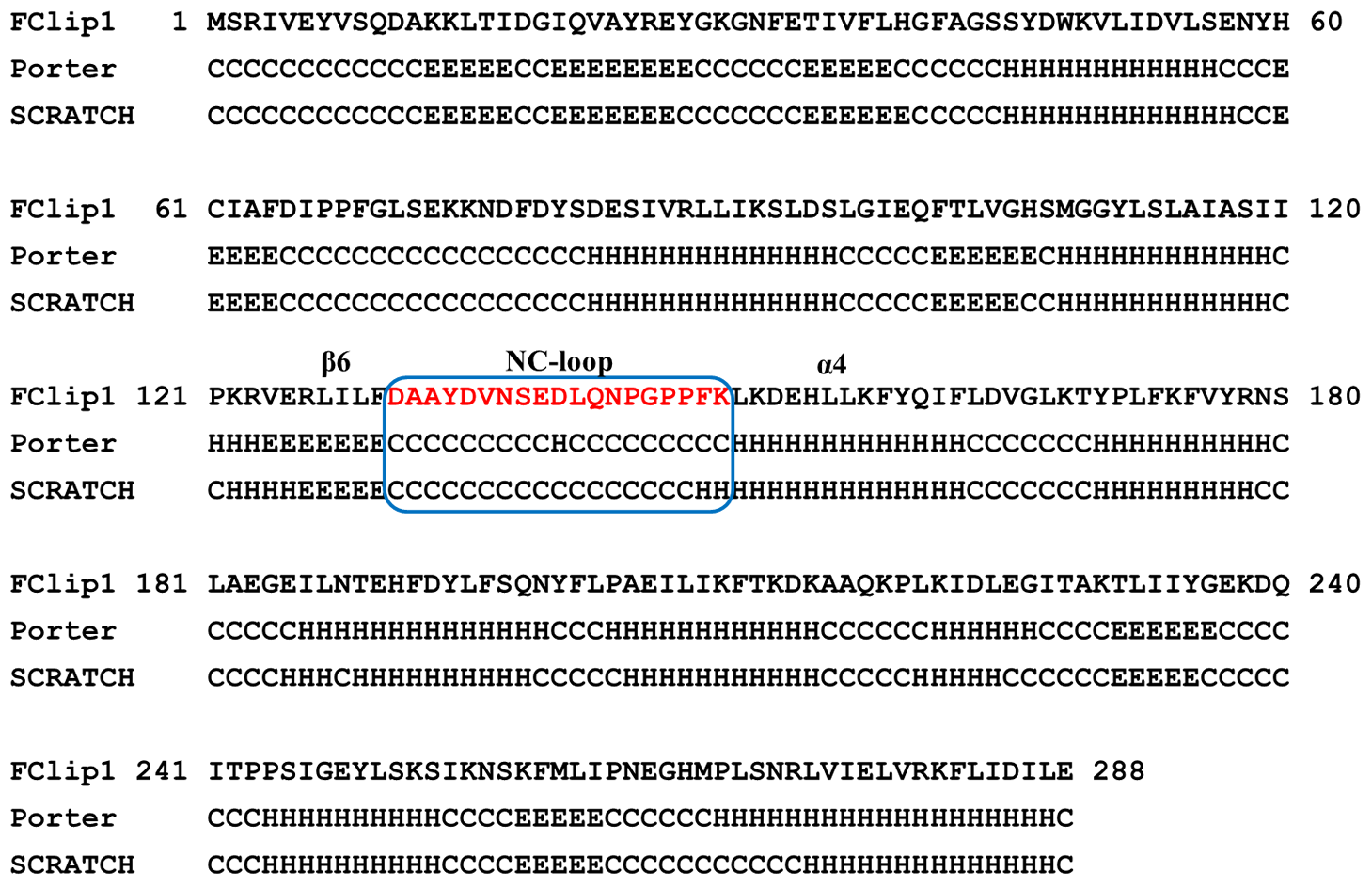

Supplement: Figure S3 — Secondary structure prediction for FClip1 by using the online servers Porter and SCRATCH. H, E, and C in the prediction results stand for the secondary structure of helix, strand and coil, respectively. The NC-loop in the modeled structure of FClip1 is shown in red. (TIF) [file pone.0046881.s003.tif]

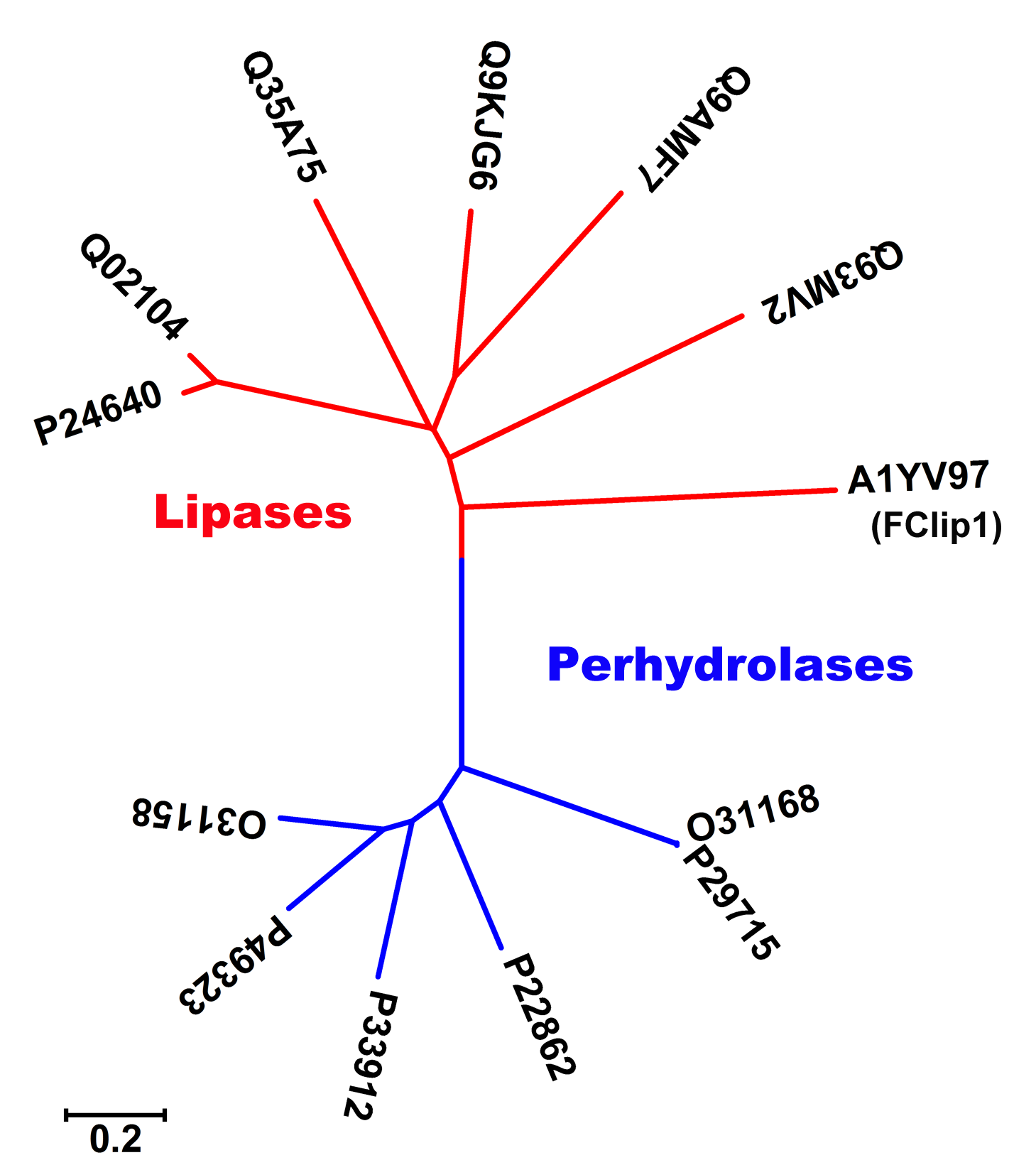

Supplement: Figure S4 — Phylogenetic tree of the lipases and the perhydrolases (non-heme haloperoxidases). P22862: aryesterase from Pseudomonas fluorescens; the other enzymes repesented by the accession numbers were the same as in figure 1. Although FClip1 (A1YV97) belongs to the lipase family, it situates at the root of the lipases branch and is near the perhydrolases branch. It might be an evolutionary intermediate between lipases and perhydrolases (Scale bar: 0.2 amino acid substitutions per site). (TIF) [file pone.0046881.s004.tif]

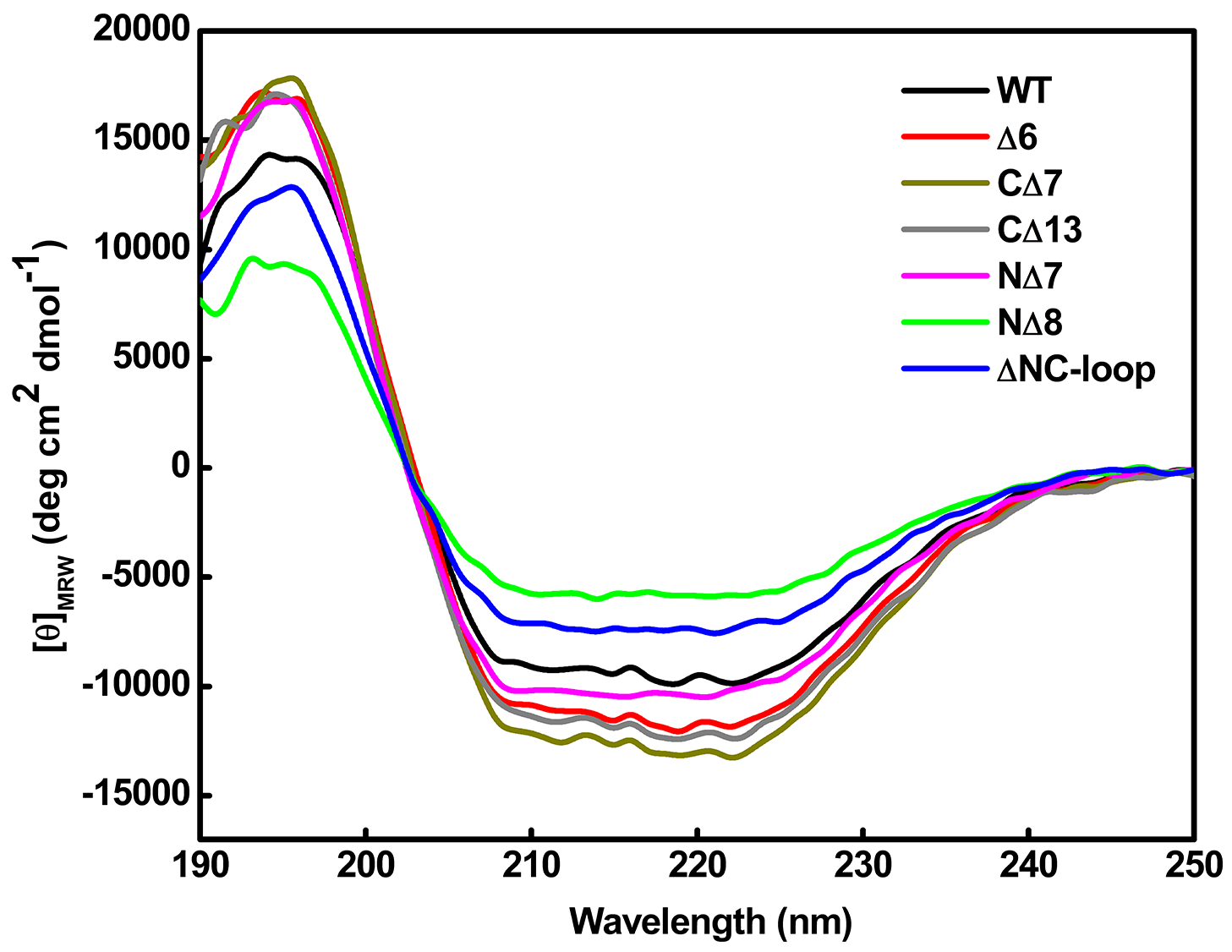

Supplement: Figure S5 — Far-UV CD spectra of the wild type FClip1 and the NC-loop deletion mutants. CD spectra were recorded from 190 to 250 nm. The spectra of wild type and the ΔNC-loop, Δ6, NΔ7, NΔ8, CΔ7, and CΔ13 mutants are shown in black, blue, red, pink, green, dark yellow, and grey, respectively. Protein concentration of 0.1 mg ml−1 was used for the analysis. (TIF) [file pone.0046881.s005.tif]

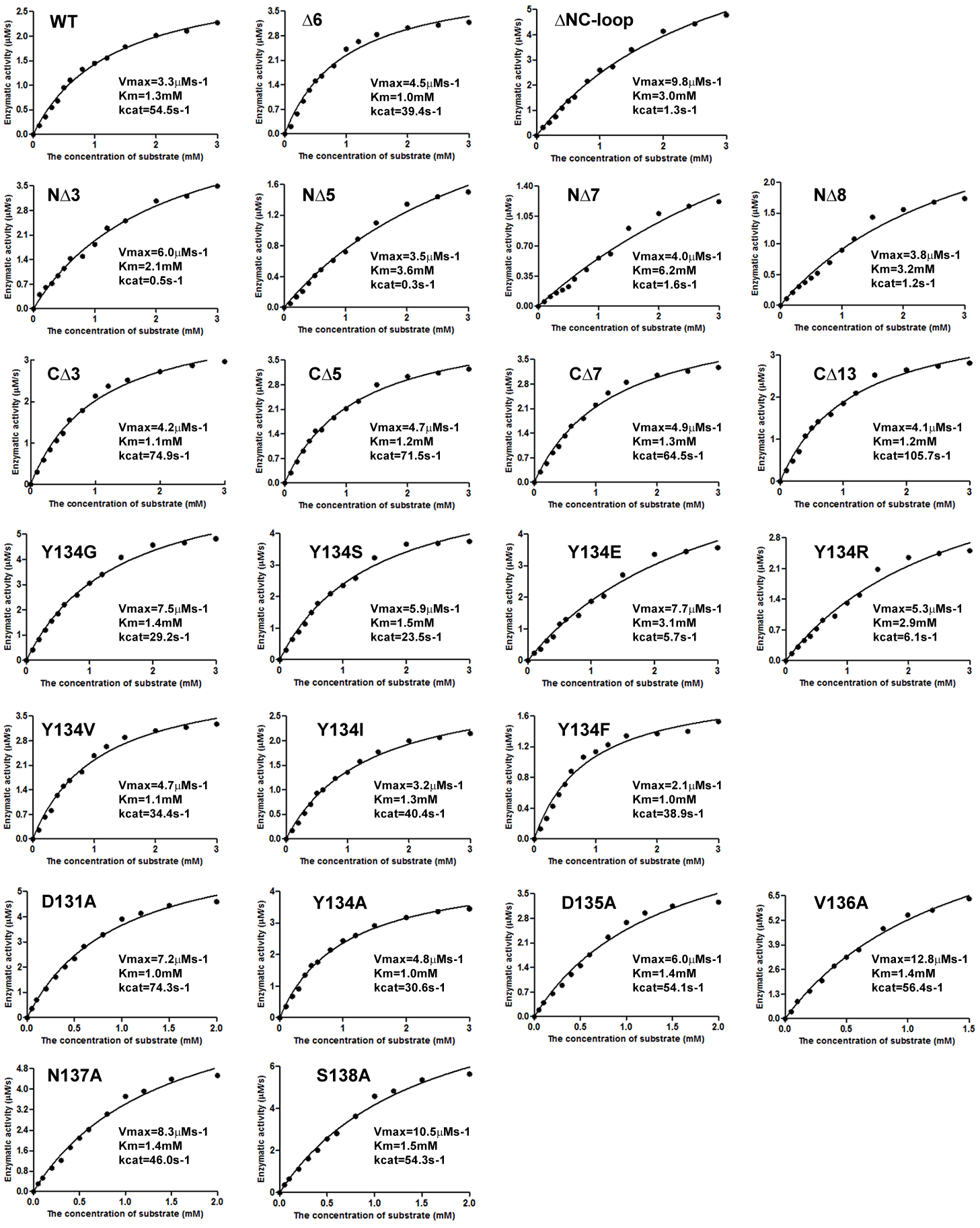

Supplement: Figure S6 — Fitting curves for the kinetic parameters from non-linear regression methods. Kinetic parameters Vmax and K m were acquired by fitting enzymatic activities as a function of substrate concentrations to the Michaelis-Menten equation using the software GraphPad Prism 5.0. (TIF) [file pone.0046881.s006.tif]

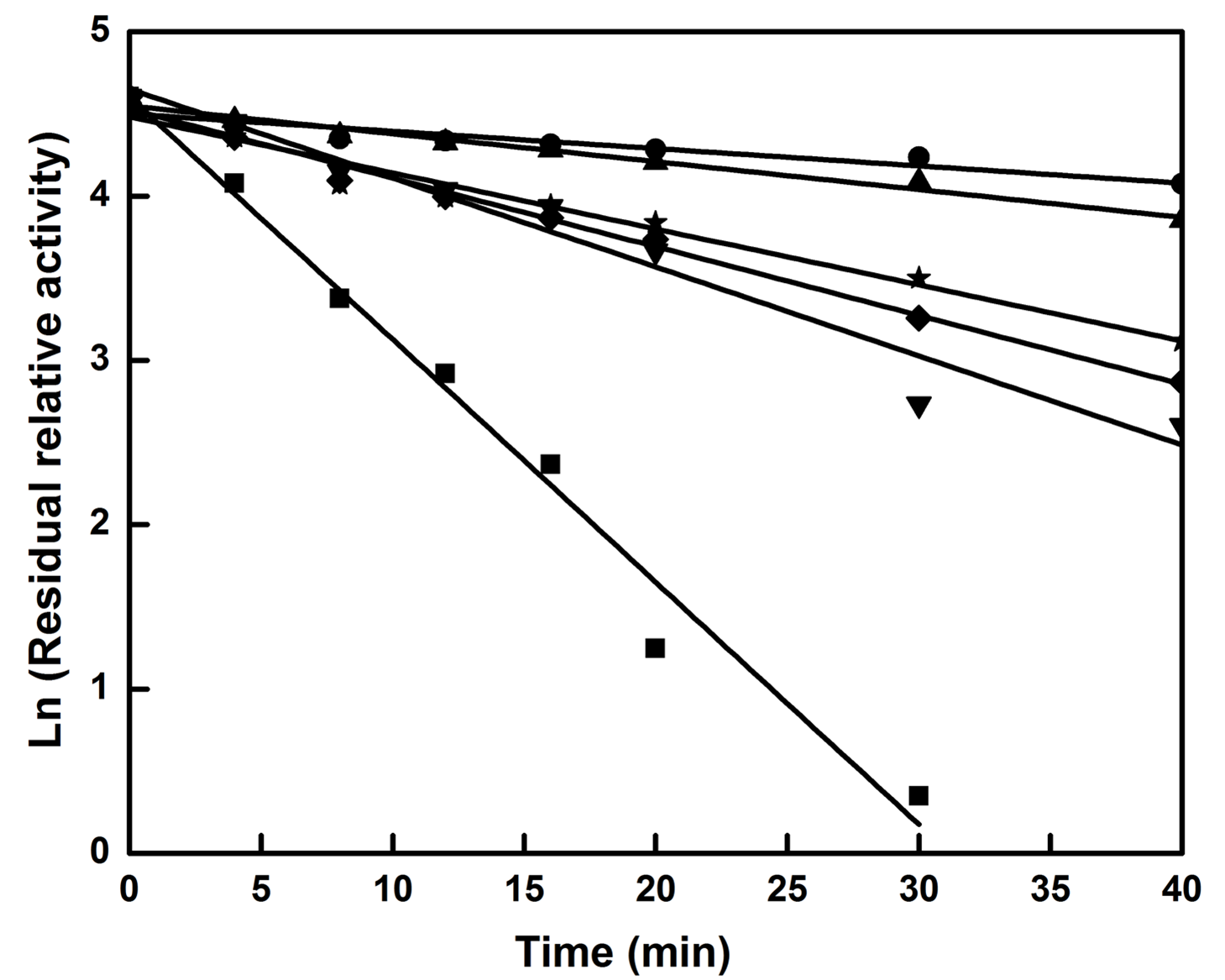

Supplement: Figure S7 — Thermal inactivation profiles of the wild type FClip1 and the NC-loop deletion mutants. After incubating the enzymes (0.5 mg ml−1) for specified time intervals at 78°C, the residual activities were measured in 50 mM phosphate buffer (pH 8.0) at 70°C using pNPC8 as the substrate. The data for wild type, the Δ6, CΔ3, CΔ5, CΔ7, and CΔ13 mutants are shown in ▪, ▾, ♦, ★, ▴, and •, respectively. (TIF) [file pone.0046881.s007.tif]

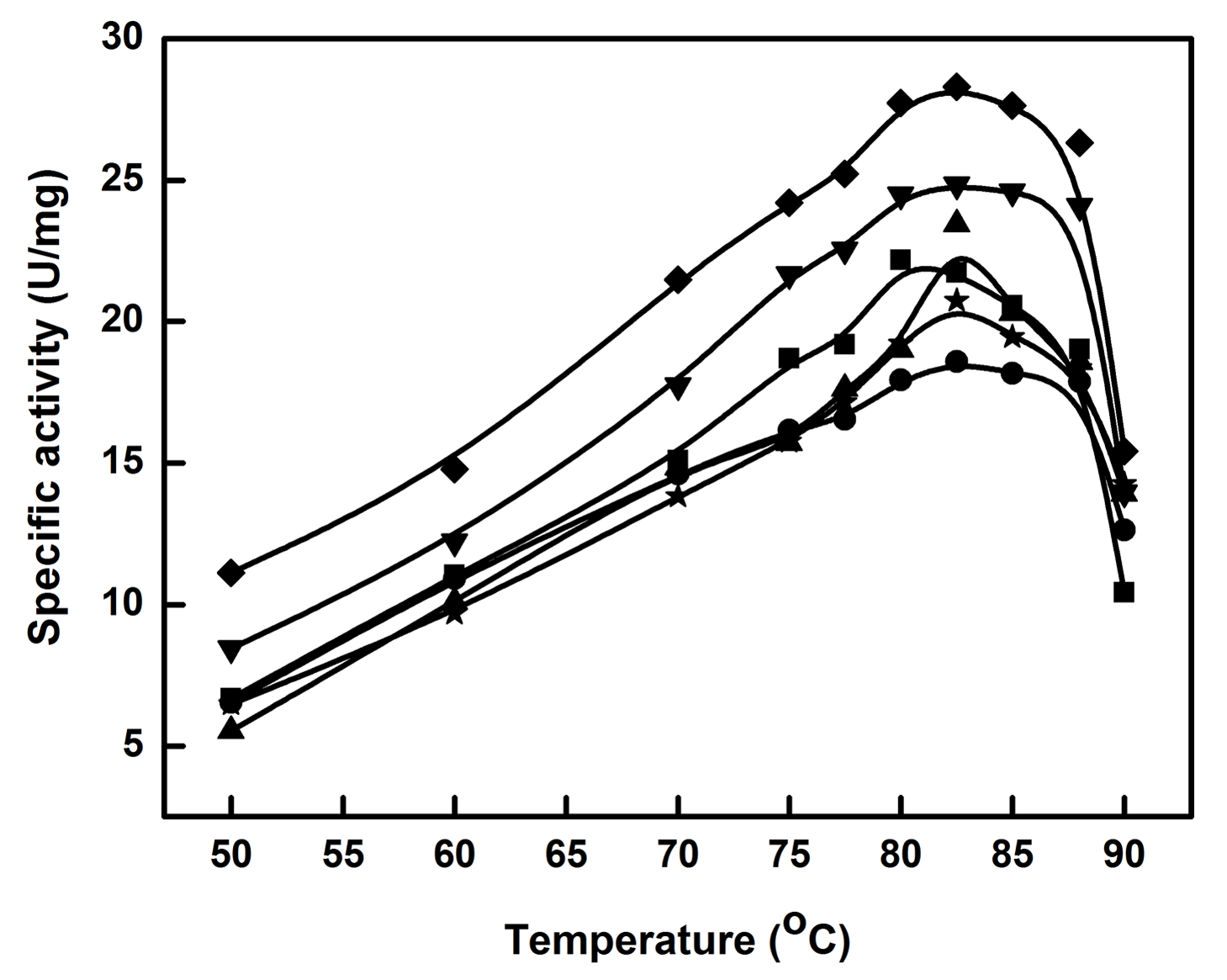

Supplement: Figure S8 — Temperature dependence of enzymatic activity of the wild type FClip1 and the NC-loop deletion mutants. Enzymatic activities were measured in the temperature range from 50 to 90°C using pNPC8 as the substrate. The data for wild type, the Δ6, CΔ3, CΔ5, CΔ7, and CΔ13 mutants are shown in ▪, ▾, ♦, ★, ▴, and •, respectively. (TIF) [file pone.0046881.s008.tif]

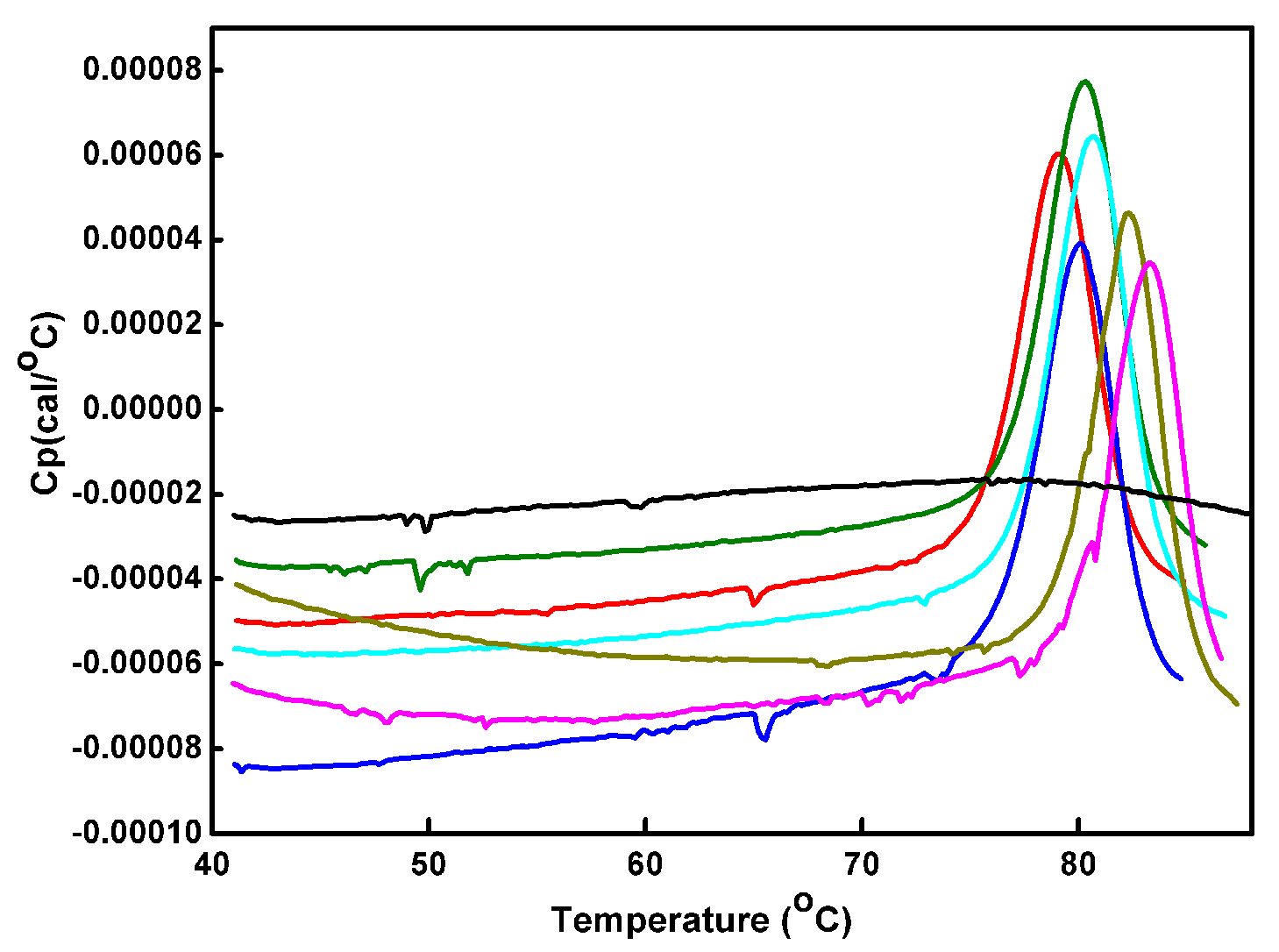

Supplement: Figure S9 — Thermal unfolding of the wild type FClip1 and the NC-loop deletion mutants monitored by DSC scanning. Protein concentration of 1.0 mg ml−1 was used for the analysis. Denaturizing curves were recorded from 40°C to 90°C with a rate of 1°C min−1. The data for baseline, wild type, and the Δ6, CΔ3, CΔ5, CΔ7, and CΔ13 mutants are shown in black, red, blue, olive, cyan, dark yellow, and pink, respectively. (TIF) [file pone.0046881.s009.tif]

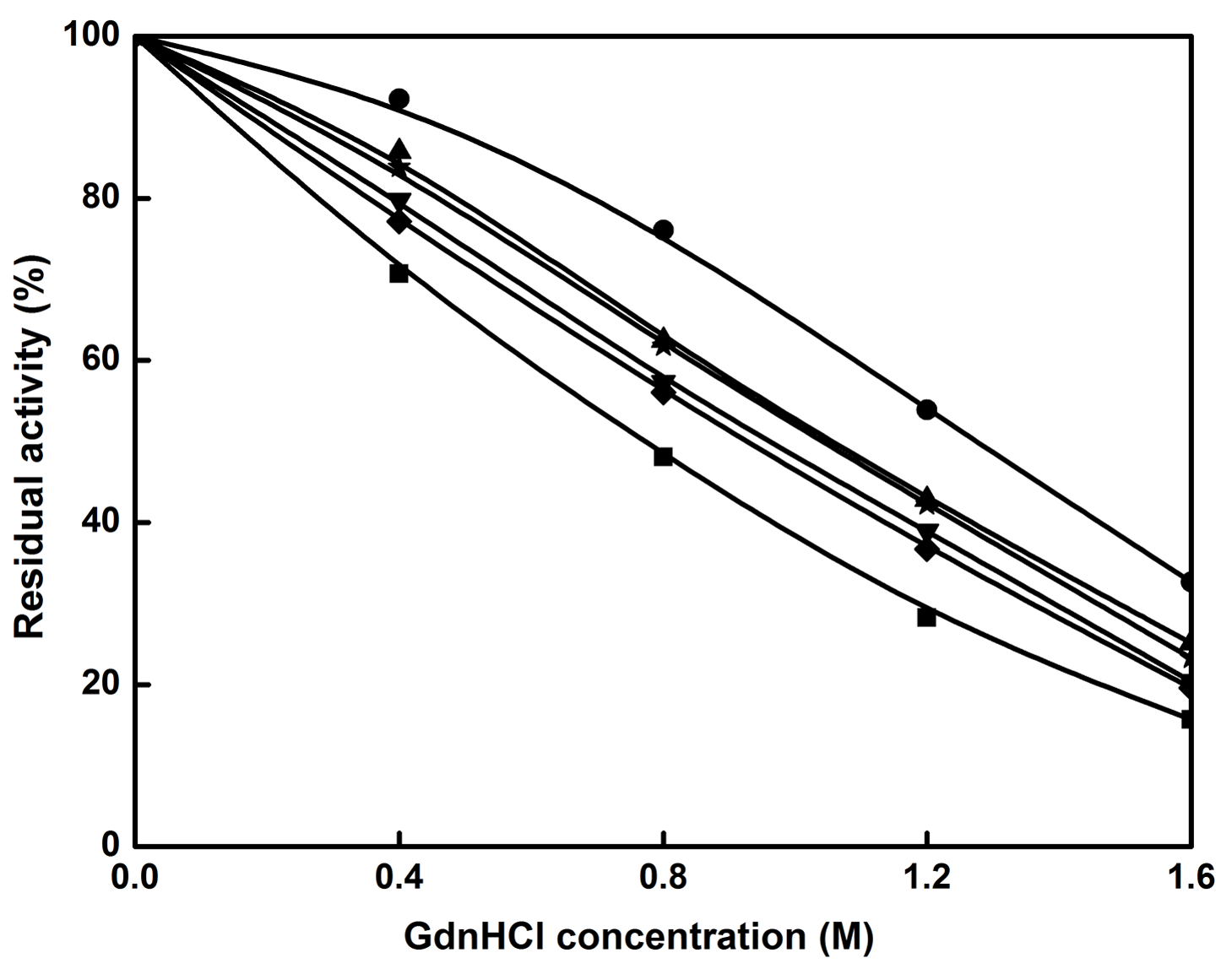

Supplement: Figure S10 — GdnHCl-induced inactivation of the wild type FClip1 and the NC-loop deletion mutants. After 2 h of incubation with varied concentrations of GdnHCl at room temperature, the residual activities were measured in 50 mM phosphate buffer (pH 8.0) at 70°C using pNPC8 as the substrate. The data for wild type, the Δ6, CΔ3, CΔ5, CΔ7, and CΔ13 mutants are shown in ▪, ▾, ♦, ★, ▴, and •, respectively. (TIF) [file pone.0046881.s010.tif]

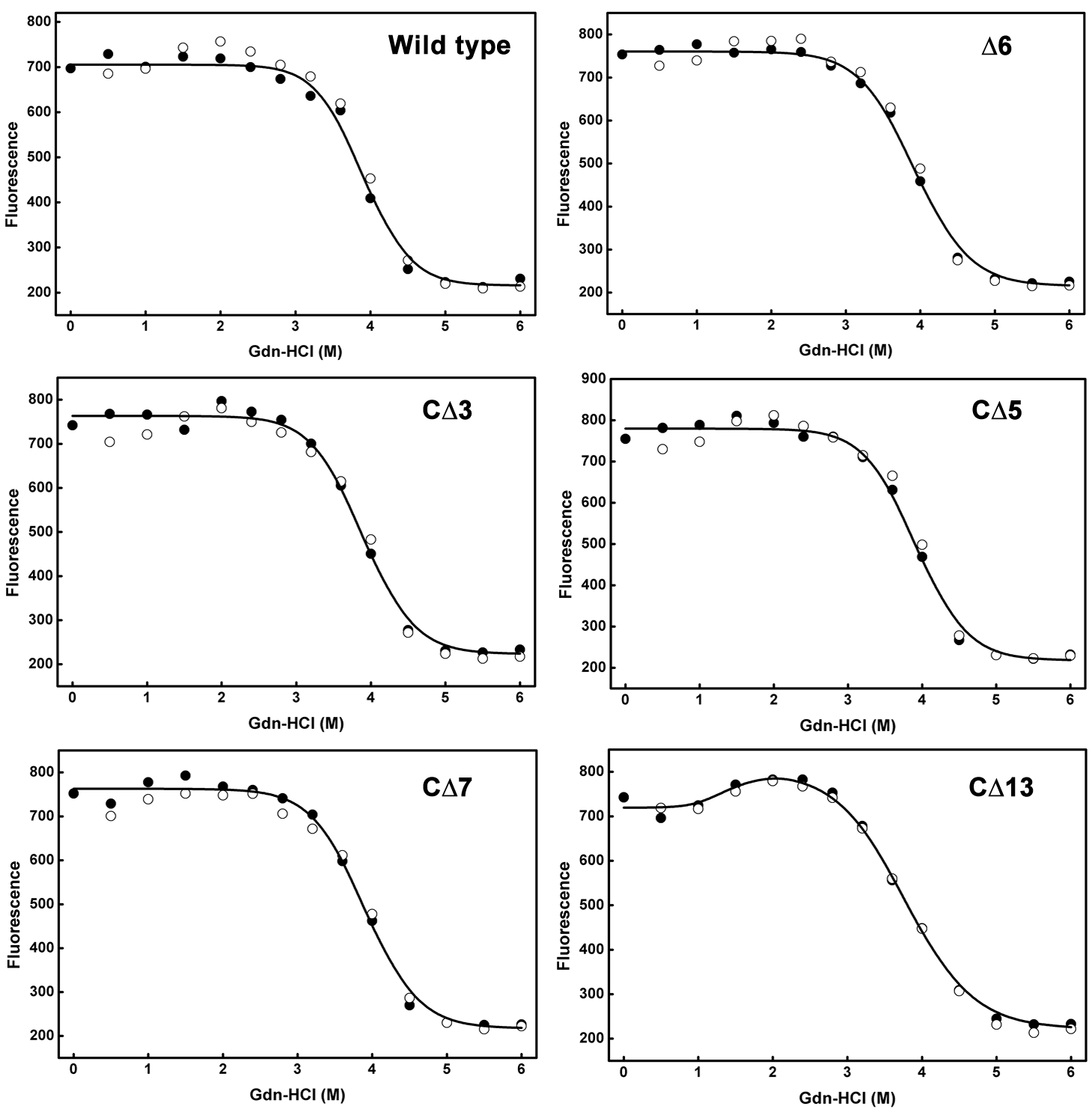

Supplement: Figure S11 — GdnHCl-induced unfolding and refolding of the wild type FClip1 and the NC-loop deletion mutants monitored by fluorescence spectrophotometry. The fluorescence spectra were recorded at the wavelengths between 300 and 400 nm with an excitation wavelength at 290 nm under a scanning speed of 1200 nm min−1. The measured data for protein unfolding and refolding are shown in • and ○, respectively. The fitted curves for protein unfolding were shown in solid lines. (TIF) [file pone.0046881.s011.tif]
