# Supplementary material for: Role of the NC-Loop in Catalytic Activity and Stability in Lipase from Fervidobacterium changbaicum
Source: PLoS One. 2012 Oct 8;7(10):e46881. doi: 10.1371/journal.pone.0046881 (PMC3466181; doi:10.1371/journal.pone.0046881)
Supplement: Table S1 — Structural modeling information of FClip1 generated by the Phyre 2 server using different templates. The qualities of the structures were evaluated by Protein Structure Validation Software (PSVS). (DOCX) [file pone.0046881.s012.docx]

**Table S1.** Structural modeling of FClip1 generated by the Phyre 2 server using different protein templates. The qualities of the structures were evaluated by Protein Structure Validation Software (PSVS).

| Template | Enzyme family | Sequence length | Sequence identity | Ramachandran plot summary | | | |
| --- | --- | --- | --- | --- | --- | --- | --- |
|  |  |  |  | Most favored regions | Additionally allowed regions | Generously allowed regions | Disallowed regions |
| 1VA4 | Haloperoxidase | 271 | 21% | 87.1% | 10.4% | 2.1% | 0.4% |
| 1A88 | Haloperoxidase | 275 | 19% | 88.7% | 9.2% | 1.7% | 0.4% |
| 1A8S | Haloperoxidase | 273 | 19% | 87.9% | 10.8% | 0.4% | 0.8% |
| 1Y37 | Fluoroacetate dehalogenase | 304 | 16% | 88.3% | 8.5% | 2.4% | 0.8% |
| 2OG1* | Carbon-carbon bond hydrolase | 286 | 23% | 86.9% | 10.7% | 2.0% | 0.4% |
| 1EHY* | Epoxide hydrolase | 293 | 19% | 85.2% | 10.4% | 3.7% | 0.4% |
| 1BN7* | Haloalkane dehalogenase | 291 | 19% | 85.9% | 12.0% | 1.2% | 0.8% |
| 1UK8* | Carbon-carbon bond hydrolase | 271 | 21% | 87.9% | 9.4% | 1.3% | 1.3% |
| 2R11* | Carboxylesterase | 306 | 17% | 88.8% | 8.2% | 1.3% | 1.7% |
| 1J1I* | Carbon-carbon bond hydrolase | 268 | 19% | 89.2% | 8.1% | 0.9% | 1.8% |

* The structures contain some invisible residues in the NC-loop region and therefore are excluded for further analysis.
