# Supplementary material for: Role of the NC-Loop in Catalytic Activity and Stability in Lipase from Fervidobacterium changbaicum
Source: PLoS One. 2012 Oct 8;7(10):e46881. doi: 10.1371/journal.pone.0046881 (PMC3466181; doi:10.1371/journal.pone.0046881)
Supplement: Table S2 — Secondary structure analysis of the wild type FClip1 and the deleted mutants by CD spectra and structural modeling. (DOCX) [file pone.0046881.s013.docx]

**Table S2.** Secondary structure analysis of the wild type FClip1 and the deleted mutants by CD spectra and structural modeling.

|  |  | α-helix | β-sheet | Turns + unstructured |
| --- | --- | --- | --- | --- |
| CD spectroscopy | WT | 32% | 20% | 48% |
|  | ∆NC-loop | 24% | 26% | 50% |
|  | ∆6 | 37% | 15% | 48% |
|  | N∆7 | 35% | 17% | 48% |
|  | N∆8 | 21% | 28% | 51% |
|  | C∆7 | 40% | 12% | 48% |
|  | C∆13 | 38% | 14% | 48% |
| Structural modeling | WT | 40% | 17% | 43% |
|  | C∆13 | 42% | 18% | 40% |

Secondary structure composition from CD spectra was calculated by using the software CDPro (CONTINLL, reference protein set: SMP56).
