# Supplementary material for: Role of the NC-Loop in Catalytic Activity and Stability in Lipase from Fervidobacterium changbaicum
Source: PLoS One. 2012 Oct 8;7(10):e46881. doi: 10.1371/journal.pone.0046881 (PMC3466181; doi:10.1371/journal.pone.0046881)
Supplement: Table S3 — Oligonucleotide primers used in the mutagenic PCR for deletion mutagenesis and site-directed mutagenesis of FClip1. (DOCX) [file pone.0046881.s014.docx]

**Table S3.** Oligonucleotide primers used in the mutagenic PCR for deletion mutagenesis and site-directed mutagenesis of FClip1.

| Mutants | Primer name | Nucleotide sequence (5’ to 3’ ) |
| --- | --- | --- |
| ∆NC-loop | F | ATTTTATTTGACGAGCACCTACTTAAGTTTTAC |
|  | R | GTGCTCGTCAAATAAAATGAGCCTTTCCACTCT |
| ∆6 | F | GATTTACAAAAGCTAAAAGACGAGCACCTAC |
|  | R | TTTTAGCTTTTGTAAATCTTCACTGTTAAC |
| N∆3 | F | ATTTTATTTTACGATGTTAACAGTGAAG |
|  | R | AACATCGTAAAATAAAATGAGCCTTTCC |
| N∆5 | F | ATTTTATTTGTTAACAGTGAAGATTTAC |
|  | R | ACTGTTAACAAATAAAATGAGCCTTTCC |
| N∆7 | F | ATTTTATTTAGTGAAGATTTACAAAAC |
|  | R | ATCTTCACTAAATAAAATGAGCCTTTC |
| N∆8 | F | ATTTTATTTGAAGATTTACAAAACCCAG |
|  | R | TAAATCTTCAAATAAAATGAGCCTTTCC |
| C∆3 | F | CCTCCGTTTGACGAGCACCTACTTAAGTTTTAC |
|  | R | GTGCTCGTCAAACGGAGGGCCTGGGTTTTGTA |
| C∆5 | F | CCAGGCCCTGACGAGCACCTACTTAAGTTTTAC |
|  | R | GTGCTCGTCAGGGCCTGGGTTTTGTAAATCTT |
| C∆7 | F | CAAAACCCAGACGAGCACCTACTTAAGTTTTACC |
|  | R | GTGCTCGTCTGGGTTTTGTAAATCTTCACTGTT |
| C∆13 | F | GTTAACAGTGACGAGCACCTACTTAAGTTTTAC |
|  | R | AGGTGCTCGTCACTGTTAACATCGTACGCAGC |
| Y134F | F | TGACGCTGCGTTCGATGTTAACAGTGAAG |
|  | R | GTTAACATCGAACGCAGCGTCAAATAAAAT |
| Y134I | F | TGACGCTGCGATCGATGTTAACAGTGAAG |
|  | R | GTTAACATCGATCGCAGCGTCAAATAAAAT |
| Y134V | F | TGACGCTGCGGTCGATGTTAACAGTGAAG |
|  | R | GTTAACATCGACCGCAGCGTCAAATAAAAT |
| Y134S | F | TGACGCTGCGTCCGATGTTAACAGTGAAG |
|  | R | GTTAACATCGGACGCAGCGTCAAATAAAAT |
| Y134G | F | TGACGCTGCGGGCGATGTTAACAGTGAAG |
|  | R | GTTAACATCGCCCGCAGCGTCAAATAAAAT |
| Y134R | F | TGACGCTGCGCGCGATGTTAACAGTGAAG |
|  | R | GTTAACATCGCGCGCAGCGTCAAATAAAAT |
| Y134E | F | TGACGCTGCGGAGGATGTTAACAGTGAAG |
|  | R | GTTAACATCCTCCGCAGCGTCAAATAAAAT |
| D131A | F | TTTATTTGCCGCTGCGTACGATGTTAACAGTGA |
|  | R | CATCGTACGCAGCGGCAAATAAAATGAGCCTT |
| Y134A | F | TGACGCTGCGGCCGATGTTAACAGTGAAG |
|  | R | GTTAACATCGGCCGCAGCGTCAAATAAAAT |
| D135A | F | GCTGCGTACGCTGTTAACAGTGAAGATTT |
|  | R | ACTGTTAACAGCGTACGCAGCGTCAAATA |
| V136A | F | GCGTACGATGCTAACAGTGAAGATTTACAA |
|  | R | TTCACTGTTAGCATCGTACGCAGCGTCAAA |
| N137A | F | TACGATGTTGCCAGTGAAGATTTACAAAACCC |
|  | R | AATCTTCACTGGCAACATCGTACGCAGCGTCA |
| S138A | F | GATGTTAACGCTGAAGATTTACAAAACCCAGG |
|  | R | GTAAATCTTCAGCGTTAACATCGTACGCAGC |

The underlined bases designate the codons where the mutations are introduced. Both forward (F) and reverse (R) primers are shown.
